# Laparoscopic para-aortic and pelvic lymphadenectomy before chemoradiotherapy was not associated with improved survival in patients with FIGO stage IIB–IIIC cervical cancer: a single-center retrospective study

**DOI:** 10.3389/fonc.2026.1975884

**Published:** 2026-10-07

**Authors:** Qin Zhang, Lin Li, Xiuying Li, Dongling Zou

**Affiliations:** Department of Gynecologic Oncology, Chongqing University Cancer Hospital & Chongqing Cancer Institute & Chongqing Cancer Hospital, Chongqing, China

**Keywords:** cervical cancer, chemoradiotherapy, lymph nodes, overall survival, progression-free survival, surgical staging

## Abstract

**Background:**

Currently, whether surgical staging can confer a survival benefit in patients with cervical cancer remains controversial, and its efficacy is similarly uncertain across different subgroups. The primary aim of this study was to assess the efficacy of laparoscopic para-aortic and pelvic lymphadenectomy prior to chemoradiotherapy in patients with International Federation of Gynecology and Obstetrics (FIGO) stage IIB–IIIC cervical cancer. The secondary aim was to identify the main prognostic factors.

**Methods:**

A total of 396 patients were included in this retrospective study, 97 patients who underwent laparoscopic para-aortic and pelvic lymphadenectomy before chemoradiotherapy comprised the surgical staging group, and 299 patients who received chemoradiotherapy alone comprised the clinical staging group. Kaplan-Meier and log-rank methods were used to construct and compare progression-free survival (PFS) and overall survival (OS) curves, respectively. Multivariable Cox proportional hazards regression models were used to identify independent prognostic factors.

**Results:**

There were no statistically significant differences in PFS (*p* = 0.187) or OS (*p* = 0.490) between the two groups. The estimated 5-year PFS rates were 67.0% (95%CI, 56.9%–75.7%) in the surgical staging group and 73.6% (95%CI, 67.4%–78.7%) in the clinical staging group. The corresponding 5-year OS rates were 76.3% (95%CI, 66.0%–83.9%) and 78.6% (95%CI, 72.7%–83.2%), respectively. Subgroup analysis stratified by imaging lymph node status demonstrated comparable survival outcomes. Multivariate analysis identified adenocarcinoma, pelvic wall involvement, and imaging-positive lymph nodes as independent risk factors for PFS; adenocarcinoma, adenosquamous carcinoma, pelvic wall involvement, and imaging-positive lymph nodes were independent predictors of OS.

**Conclusions:**

Laparoscopic para-aortic and pelvic lymphadenectomy before chemoradiotherapy was not associated with improved PFS or OS in this retrospective cohort, Tumor histology, pelvic wall involvement, and imaging-positive lymph nodes were the main prognostic factors.

## Introduction

Cervical cancer is the fourth most common cancer among women in terms of both incidence and mortality, with an estimated 660,000 new cases and 350,000 deaths worldwide in 2022 (1). In China, approximately 151,000 new cervical cancer cases and 56,000 deaths occurred in 2022 (2). Lymph node metastasis is one of the most important prognostic factors associated with poor survival in patients with cervical cancer (3–5), and patients with para-aortic lymph node metastasis have a significantly worse prognosis than those with pelvic lymph node metastasis (6). The most important update in the 2018 International Federation of Gynecology and Obstetrics (FIGO) staging system was the formal integration of imaging and pathological lymph node status into clinical staging, together with the newly defined Stage IIIC for lymph node metastasis. Accordingly, a proportion of patients previously classified as Stage I–II are upstaged to Stage IIIC under the revised criteria, and their standard treatment strategy shifts from radical surgery to chemoradiotherapy. For locally advanced cervical cancer, concurrent chemoradiotherapy remains the standard first-line treatment (7). Concurrent chemoradiotherapy comprises external beam radiotherapy (EBRT), brachytherapy, and concurrent platinum-containing chemotherapy. EBRT is delivered to the pelvis with or without the para-aortic region depending on common iliac and/or para-aortic lymph node metastasis. Extended-field EBRT (EF-EBRT) is recommended when imaging or pathology confirms involvement of the common iliac or para-aortic lymph nodes; therefore, accurate identification of lymph node metastasis is essential for treatment planning and prognostic stratification.

Magnetic resonance imaging (MRI), computed tomography (CT), and positron emission tomography–computed tomography (PET-CT) are the primary imaging modalities used to detect lymph node metastasis in patients with cervical cancer. Although these modalities offer relatively high sensitivity and specificity, their diagnostic accuracy remains insufficient to replace histopathologic confirmation. Patients with International Federation of Gynecology and Obstetrics (FIGO) stage IIICr cervical cancer may have pathologically negative lymph nodes (8), whereas imaging-negative lymph nodes may harbor pathologically confirmed metastases (9, 10). Studies have confirmed that patients with pelvic lymph node metastasis have a higher incidence of para-aortic lymph node metastasis (8, 11).

Surgical staging can accurately diagnose lymph node metastasis through pathologic examination; however, whether it confers a survival benefit in patients with cervical cancer remains controversial. Some studies have demonstrated that surgical staging before chemoradiotherapy does not improve survival outcomes in patients with cervical cancer. In a prospective study by Simone Marnitz et al., 255 patients with locally advanced cervical cancer were randomly assigned (1:1) to surgical staging (para-aortic and pelvic lymphadenectomy) or clinical staging followed by primary platinum-based chemoradiation. Although no difference in disease-free survival was observed between the two groups, patients with FIGO stage IIB disease had a significant disease-free survival benefit (12). Another retrospective study also found no significant difference in 5-year disease-free survival or OS between surgical and imaging staging groups (13). Two further studies suggested that para-aortic surgical staging may adversely affect disease-free survival or OS in patients with locally advanced cervical cancer (14, 15).

Several studies have reported a survival benefit of surgical staging before chemoradiotherapy in patients with locally advanced cervical cancer, although its efficacy remains controversial across subgroups. Two studies demonstrated improved disease-free survival or OS with surgical staging in patients with FIGO stage II disease or in those without radiologic lymph node metastasis (16, 17). A study by Wei Jiang et al. reported improved 5-year disease-free survival or OS in patients with lymph node diameters ≥ 1.5 cm or ≥ 2 metastatic lymph nodes (18). Therefore, further clinical studies are needed to evaluate whether resection of the para-aortic and pelvic lymph nodes before chemoradiotherapy can improve the prognosis of patients with cervical cancer. Currently, three prospective clinical studies are evaluating the effectiveness of lymph node dissection in patients with FIGO 2018 stage IIICr cervical cancer (19–21).

This single-center retrospective cohort study enrolled patients with FIGO stage IIB–IIIC cervical cancer. Patients in the surgical staging group underwent laparoscopic para-aortic and pelvic lymphadenectomy followed by chemoradiotherapy, whereas those in the clinical staging group received chemoradiotherapy alone. We compared survival outcomes between the two groups. The primary aim was to assess whether surgical staging before chemoradiotherapy improves survival; the secondary aim was to identify the main prognostic factors.

## Methods

### Patients and treatment protocol

This retrospective study was approved by the Ethics Committee of Chongqing University Cancer Hospital (Approval No. CZLS2021042-A-051). A total of 396 patients with FIGO stage IIB–IIIC cervical cancer who completed treatment at our center between April 2014 and April 2020 were retrospectively enrolled. The data cut-off date was April 24, 2026. Treatment decisions were made through multidisciplinary team discussion and based on multiple clinical factors, including Cooperative Oncology Group performance status (ECOG PS), baseline comorbidities, imaging-based staging, surgical risk tolerance, and informed patient preference. Surgical staging was preferentially recommended for patients with good performance status and adequate surgical fitness in whom complete resection of enlarged lymph nodes was considered feasible. Patients with poor performance status, severe comorbidities precluding abdominal surgery, or those who declined surgical intervention directly received chemoradiotherapy. The surgical staging group comprised 97 patients who underwent laparoscopic para-aortic and pelvic lymphadenectomy followed by chemoradiotherapy, and the clinical staging group comprised 299 patients who received chemoradiotherapy. Inclusion criteria were as follows: (1) age ≥ 20 and ≤ 70 years; (2) ECOG PS ≤ 1; (3) newly diagnosed squamous cell carcinoma, adenocarcinoma, or adenosquamous carcinoma, with no previous antitumor therapy before staging surgery or chemoradiotherapy; (4) thorough pretreatment assessment of the tumor and lymph node involvement using CT, MRI, and/or PET-CT; and (5) FIGO 2018 stage IIB–IIIC. Exclusion criteria were as follows: (1) malignant tumors of other tissues or organs; (2) active infectious disease; (3) history of subtotal hysterectomy; (4) history of pelvic radiotherapy; (5) pretreatment hemoglobin < 70 g/L; (6) cervical cancer during pregnancy; and (7) total duration of EBRT and brachytherapy > 3 months.

Lymph nodes were classified as imaging-positive if they met one of the following criteria: short-axis diameter > 1.0 cm; markedly high signal intensity on diffusion-weighted imaging; significant morphologic changes compared with normal lymph nodes; or PET-CT SUVmax > 2.5 (22, 23). In this study, surgical staging was performed laparoscopically, with dissection of pelvic and para-aortic lymph nodes and no conversion to open surgery. The procedure involved removal of lymph nodes from the obturator, external iliac, internal iliac, common iliac, and inframesenteric para-aortic regions. In addition, all bulky or suspected metastatic lymph nodes located between the inferior mesenteric artery and the renal vein were removed. Pathology results were available within 1 week after surgery, and radiotherapy plans were formulated according to these results. Patients usually started chemoradiotherapy within 2 weeks after pathology results became available. Chemoradiotherapy comprised EBRT/EF-EBRT, brachytherapy, and/or concurrent platinum-containing chemotherapy. EBRT was delivered to the pelvis with or without the para-aortic region on the basis of iliac and/or para-aortic nodal involvement. EF-EBRT was recommended when common iliac and/or para-aortic lymph nodes were confirmed by imaging or pathologic examination. The EBRT/EF-EBRT dose was approximately 45–50.4 Gy in 25–28 fractions; positive lymph nodes could receive a boost of 10–20 Gy using reduced-volume EBRT. Brachytherapy was usually initiated after 15 EBRT fractions, at a dose of 30–42 Gy in 5–7 fractions. The cumulative dose at point A exceeded 80 Gy. Cisplatin-based chemotherapy was administered during EBRT/EF-EBRT. Weekly chemotherapy consisted of intravenous cisplatin 30–40 mg/m² administered 4–9 times, whereas triweekly chemotherapy consisted of cisplatin 60–75 mg/m² administered 2–3 times. Systemic chemotherapy was not administered after completion of radiotherapy. The flowchart of the study is shown in **Figure 1**.

**Figure 1:**
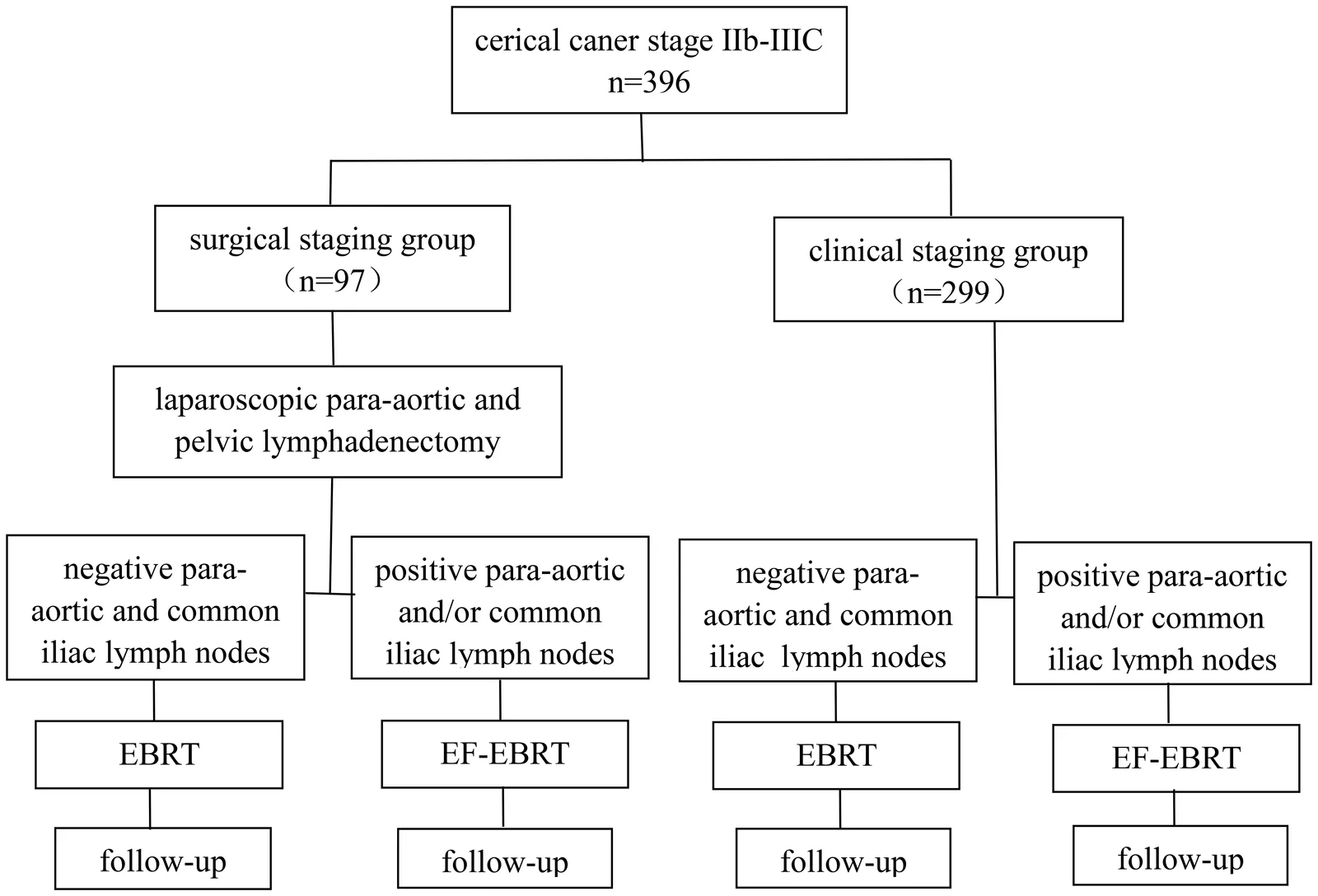
Flowchart of the study.

### Follow-up and data collection

All patients underwent routine post-treatment surveillance, including physical status assessment, gynecological examination, squamous cell carcinoma antigen testing, cervical cytology, thoracoabdominopelvic imaging, and monitoring for treatment-related complications. Baseline data and surgical information were collected by retrospective review of medical records. The vast majority of patients were followed up at our hospital; we obtained information about the patient’s survival status and survival time by reviewing the medical records. For the small proportion followed up at local hospitals, information was obtained mainly via telephone and WeChat.

The primary endpoints of the study were PFS and OS. PFS was defined as the time from the date of initial treatment to objective tumor progression or death from any cause. OS was defined as the time from the date of initial treatment to death from any cause. The data cut-off date was 2026-04-24. The censoring procedure was defined as follows: patients who were lost to follow-up or without endpoint events at the data cut-off were censored at the last known date of event-free status. Tumor recurrence was confirmed by imaging examination or pathological biopsy. Vital status was ascertained by reviewing inpatient and outpatient medical records and telephone/WeChat follow-up. Treatment-related complications were assessed according to the National Cancer Institute Common Terminology Criteria for Adverse Events (CTCAE) version 5.0. Information on grade 3/4 myelosuppression, grade 3/4 gastro-intestinal disorder, and grade 3/4 perineal and lower-extremity oedema was collected and analyzed.

### Statistical analysis

Categorical variables are expressed as frequencies and percentages, and continuous variables as the median (interquartile range) or mean ± standard deviation. Categorical variables were compared using the chi-square test or Fisher’s exact test. Survival curves were estimated using the Kaplan–Meier method and compared using the log-rank test. In the survival analyses, patients who were alive at the end of follow-up or lost to follow-up were treated as right-censored. These patients were censored at the time of their last documented follow-up. The Cox proportional hazards model handles right-censored observations via partial likelihood estimation under the assumption of noninformative censoring. Cox proportional hazards models were used to estimate hazard ratios (HRs) and 95% confidence intervals (CIs) for associations between prognostic factors and PFS or OS. Covariates for the multivariable Cox models were selected on the basis of clinical relevance and previously reported prognostic evidence, and univariable Cox results are provided only as supplementary reference. The proportional hazards assumption for the Cox regression models was assessed using Schoenfeld residuals, including graphical inspection and correlation tests. No violation of the proportional hazards assumption was identified for the primary grouping variable. Thus, results from standard Cox regression models were reported. A *p* value < 0.05 was considered statistically significant. All data were processed and analyzed using R 4.5.2.

## Results

### Baseline characteristics

The surgical staging group included 97 patients, with a median follow-up of 69.0 (range, 7.0–121.0) months, and the clinical staging group included 299 patients, with a median follow-up of 78.0 (range, 4.0–123.0) months. The baseline characteristics of the patients are presented in **Table 1**.

**Table 1:** Baseline characteristics.

Variables	Surgical staging group (n = 97)	Clinical staging group (n = 299)	*p* value	SMD
Age, years, median	51.0 (46.0–56.5)	52.0 (47.0–59.0)	0.122	0.09
ECOG PS, n (%)			0.890	0.02
0	82 (84.5)	251 (83.9)		
1	15 (15.5)	48 (16.1)		
Histologic type, n (%)			0.820	0.07
Squamous	91 (93.8)	285 (95.3)		
Adenocarcinoma	4 (4.1)	10 (3.4)		
Adenosquamous	2 (2.1)	4 (1.3)		
Tumor size, cm, n (%)			0.910	0.05
≤ 2	3 (3.1)	7 (2.4)		
2–4	31 (32.0)	94 (31.4)		
>4	63 (64.9)	198 (66.2)		
Pelvic wall involvement, n (%)			0.624	0.06
Yes	42 (43.3)	138 (46.2)		
No	55 (56.7)	161 (53.8)		
Squamous-cell carcinoma antigen, ng/mL, median	5.3 (2.6–16.2)	5.5 (2.1–16.3)	0.582	0.01
Imaging-diagnosed lymph node status, n (%)			0.892	0.02
Imaging-negative lymph nodes	44 (45.4)	138 (46.2)		
Imaging-positive pelvic and/or para-aortic lymph nodes	53 (54.6)	161 (53.8)		
Radiotherapy dose at point A, Gy, n (%)	89.0 (85.0–90.4)	85.4 (85.0–90.4)	0.482	0.04
Cisplatin-based chemotherapy, n (%)			0.621	0.06
Yes	95 (97.2)	290 (97.0)		
No	2 (2.1)	9 (3.0)		

Point A, a paracervical reference point that has been the most widely used dosimetric parameter to date. SMD, standardized mean differences.

### Characteristics of the surgical staging group

The characteristics of the surgical staging group are presented in **Table 2**. The mean number of resected lymph nodes was 35.4 ± 13.2. Postoperative pathologic results showed no para-aortic or pelvic lymph node metastasis in 47 (48.5%) patients, pelvic lymph node metastasis only in 37 (38.1%), and both para-aortic and pelvic lymph node metastasis in 13 (13.4%). No patient had isolated para-aortic lymph node metastasis without pelvic lymph node involvement. Compared with surgical pathology, preoperative imaging had a sensitivity of 80.0% and specificity of 72.3%. The median interval from surgery to chemoradiotherapy was 11.0 (9.0–13.0) days.

**Table 2:** Characteristics of the surgical staging group (n = 97).

Characteristics	Value
Number of lymph nodes removed, mean ± SD	35.4 ± 13.2
Pathologic lymph node status	
No para-aortic or pelvic lymph node metastasis, n (%)	47 (48.5%)
Pelvic lymph node metastasis only, n (%)	37 (38.1%)
Para-aortic and pelvic lymph node metastases, n (%)	13 (13.4%)
Sensitivity, %	80.0%
Specificity, %	72.3%
Median interval from surgery to chemoradiotherapy, days	11.0 (9.0–13.0)

### Survival analysis

In the surgical staging group, PFS events occurred in 33 of 97 patients (34.0%), and 26 patients (26.8%) died during follow-up. In the clinical staging group, PFS events occurred in 83 of 299 patients (27.8%), and 71 patients (23.7%) died during follow-up. Survival analyses for all patients are presented in **Figures 2A, B**. There were no statistically significant differences in PFS (*p* = 0.187) or OS (*p* = 0.490) between the two groups. The estimated 5-year PFS rates were 67.0% (95%CI, 56.9%–75.7%) for the surgical staging group and 73.6% (95%CI, 67.4%–78.7%) for the clinical staging group. The corresponding 5-year OS rates were 76.3% (95%CI, 66.0%–83.9%) and 78.6% (95%CI, 72.7%–83.2%) for the two groups, respectively.

**Figure 2:**
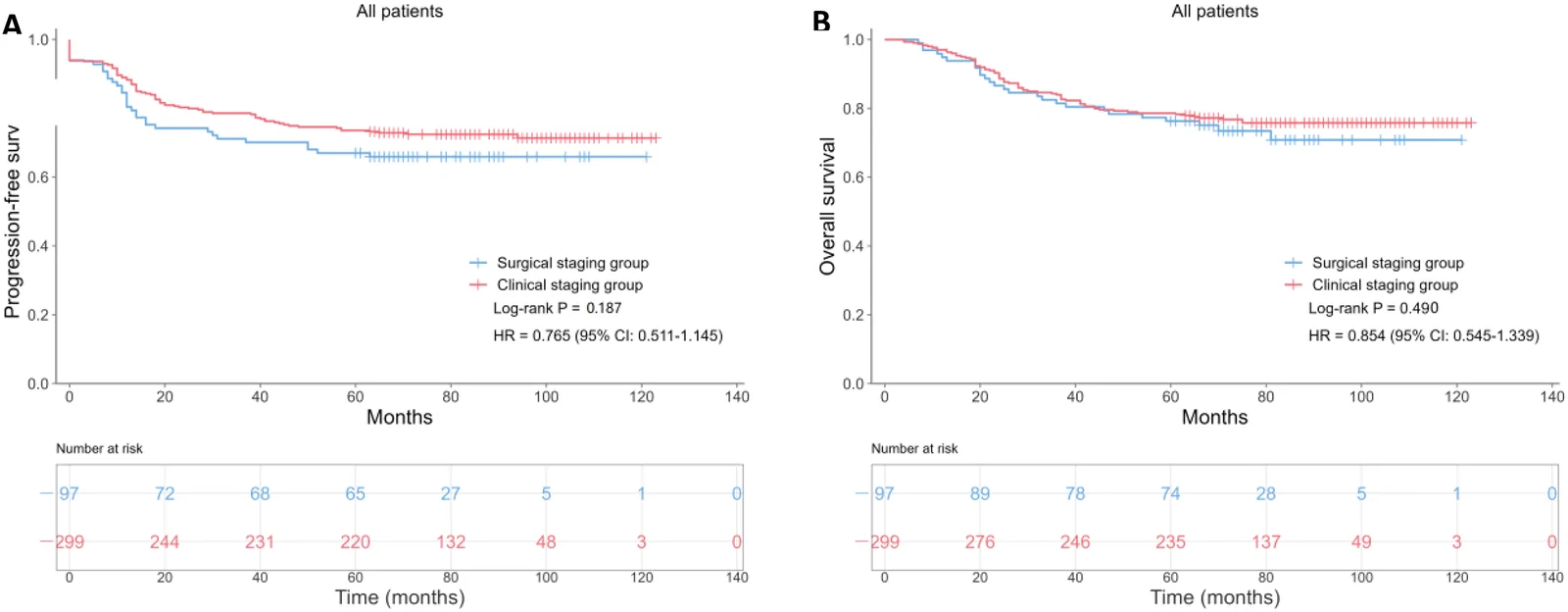
Kaplan-Meier curves for PFS **(A)** and OS **(B)** for all patients.

Patients were divided into lymph node-positive and lymph node-negative cohorts based on imaging findings for subgroup analyses. A total of 53 patients in the surgical staging group and 161 in the clinical staging group were included in the lymph node-positive cohort. Within this cohort, no statistically significant differences were observed in PFS (*p* = 0.057) or OS (*p* = 0.525). The estimated 5-year PFS rates were 50.9% (95%CI, 36.8%–63.4%) and 64.0% (95%CI, 57.9%–70.6%) for the surgical-staging and clinical-staging groups, respectively. The corresponding 5-year OS rates were 64.2% (95%CI, 49.7%–76.1%) and 68.3% (95%CI, 60.6%–74.8%) for the two groups, respectively. Survival curves for this cohort are shown in **Figures 3A, B**.

**Figure 3:**
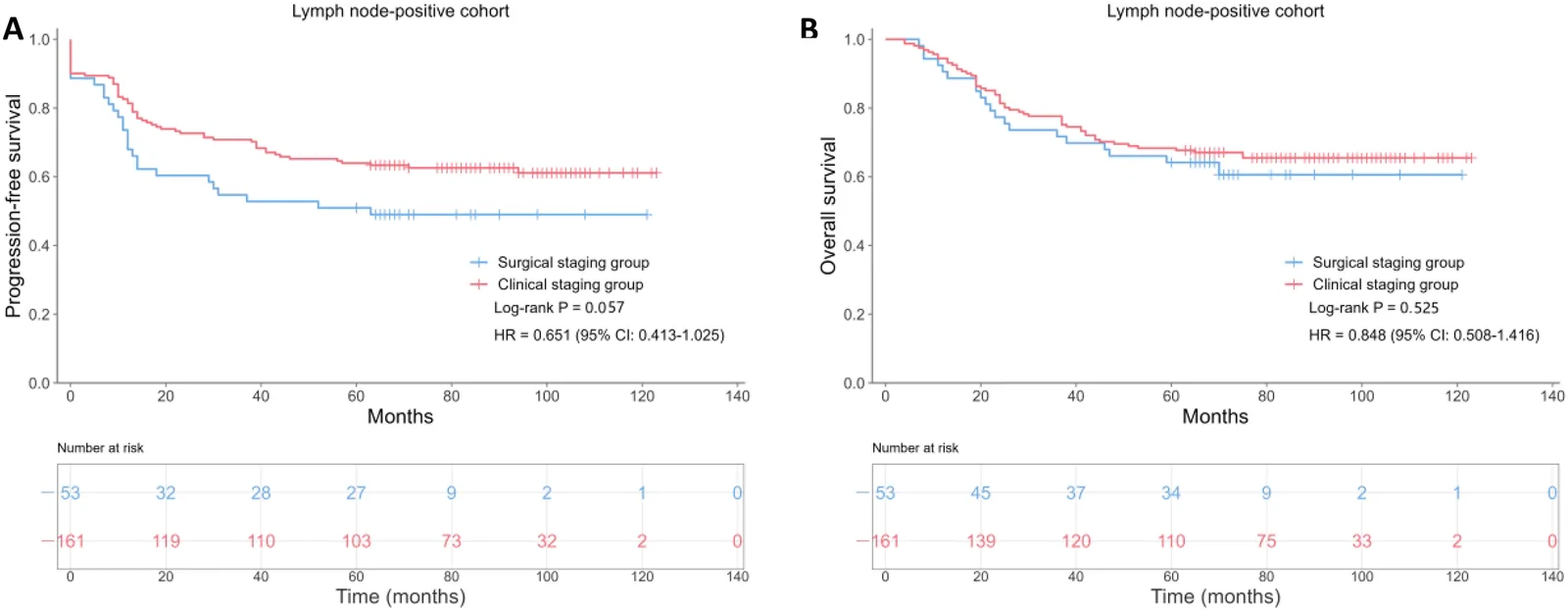
Kaplan-Meier curves for PFS **(A)** and OS **(B)** for the lymph node-positive cohort.

A total of 44 patients in the surgical staging group and 138 in the clinical staging group were included in the lymph node-negative cohort. No statistically significant differences were observed in PFS (*p* = 0.720) or OS (*p* = 0.747). The estimated 5-year PFS rates were 86.4% (95%CI, 62.0%–95.7%) and 84.8% (95%CI, 77.8%–89.8%) for the surgical and clinical staging groups, respectively. The corresponding 5-year OS rates were 90.9% (95%CI, 77.7%–96.5%) and 90.6% (95%CI, 84.4%–94.4%) for the two groups, respectively. Survival curves for this cohort are shown in **Figures 4A, B**.

**Figure 4:**
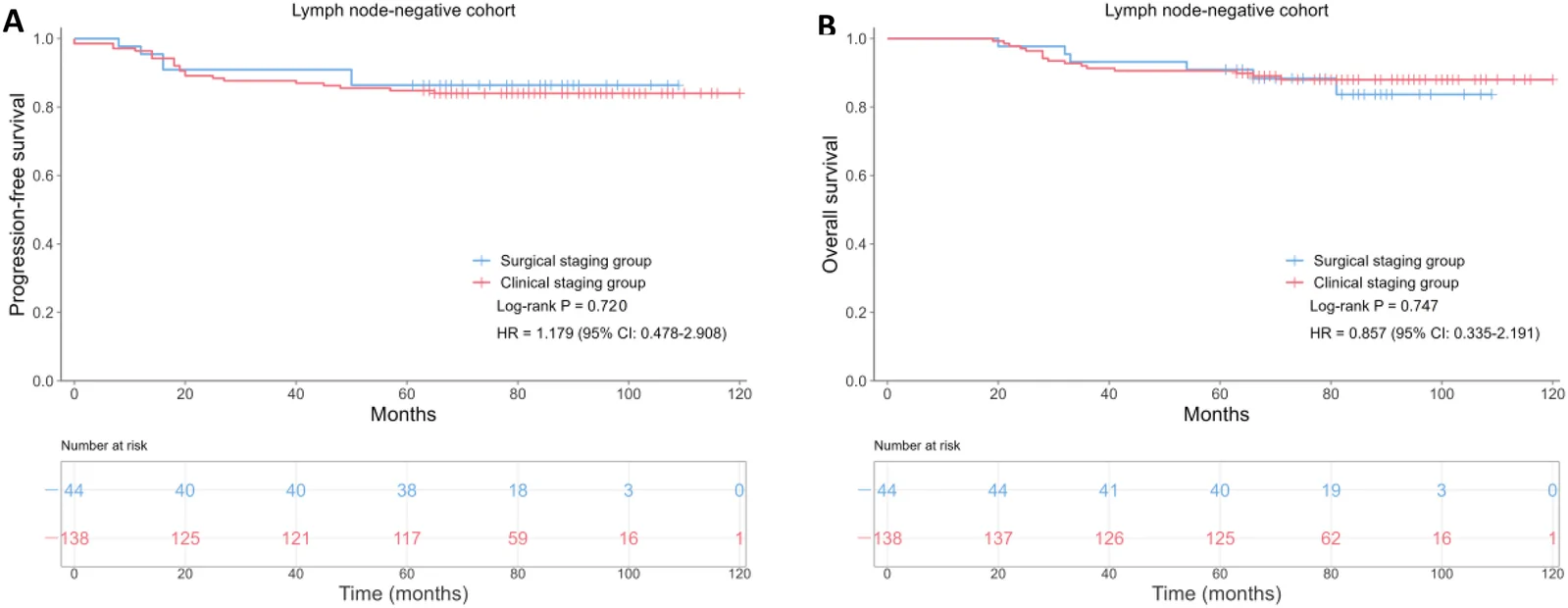
Kaplan-Meier curves for progression-free survival **(A)** and overall survival **(B)** for the lymph node-negative cohort.

### Analysis of prognostic factors

Univariate and multivariate analyses of prognostic factors are presented in **Table 3**. The proportional hazards assumption was assessed for each covariate in the univariable and multivariable Cox proportional-hazards models using scaled Schoenfeld residuals and the Grambsch–Therneau test. In the univariable analyses, no evidence of violation of the proportional hazards assumption was observed for the primary grouping variable in either the PFS or OS model (*P* = 0.616 and *P* = 0.816, respectively). After multivariable adjustment for the prespecified clinical covariates, the grouping variable likewise showed no evidence of non-proportionality in the PFS or OS model (*P* = 0.855 and *P* = 0.666, respectively). Univariate analyses showed that histologic type, pelvic wall involvement, elevated squamous-cell carcinoma antigen, and imaging-positive lymph nodes were significantly associated with PFS and OS. Multivariate analysis identified adenocarcinoma, pelvic wall involvement, and imaging-positive lymph nodes as independent risk factors for PFS. Among all factors, adenocarcinoma, adenosquamous carcinoma, pelvic wall involvement and imaging-positive lymph nodes were independent predictors of OS.

**Table 3:** Univariate and multivariate analyses of prognostic factors affecting PFS and OS.

	Univariate analysis				Multivariable analysis			
Variables	PFS		OS		PFS		OS	
	HR (95% CI)	*P* value	HR (95% CI)	*P* value	HR (95% CI)	*P* value	HR (95% CI)	*P* value
Age	1.001 (0.977-1.026)	0.921	1.000 (0.973-1.027)	0.986	1.003 (0.976-1.030)	0.854	1.004 (0.975-1.034)	0.771
ECOG PS	0.632 (0.355-1.125)	0.119	0.719 (0.393-1.316)	0.285	0.604 (0.335-1.089)	0.094	0.698 (0.375-1.298)	0.256
Histologic type	1.694 (1.076-2.668)	0.023	1.844 (1.145-2.970)	0.012				
Squamous					reference		reference	
Adenocarcinoma					2.665 (1.211-5.865)	0.015	2.375 (1.017-5.549)	0.046
Adenosquamous					2.853 (0.846-9.628)	0.091	3.653 (1.014-13.157)	0.047
Tumor Size	1.244 (0.868-1.783)	0.235	1.423 (0.946-2.141)	0.090				
≤2 cm					reference		reference	
>2 cm ≤4 cm					1.294 (0.290-5.783)	0.736	1.120 (0.230-5.463)	0.888
>4 cm					1.225 (0.279-5.379)	0.788	1.334 (0.280-6.349)	0.718
Pelvic wall involved	2.276 (1.566-3.309)	<0.001	2.391 (1.582-3.611)	<0.001	1.826 (1.222-2.730)	0.003	1.861 (1.192-2.906)	0.006
Squamous-cell carcinoma antigen	1.006 (1.000-1.011)	0.034	1.006 (1.000-1.011)	0.048	1.002 (0.997-1.008)	0.410	1.002 (0.996-1.008)	0.486
Imaging-positive pelvic and/or paraaortic lymph nodes	3.200 (2.090-4.898)	<0.001	3.416 (2.123-5.496)	<0.001	2.956 (1.889, 4.627)	<0.001	2.963 (1.798-4.849)	<0.001
Radiotherapy dose at point A	0.990 (0.953-1.029)	0.612	0.978 (0.942-1.015)	0.233	0.981 (0.942-1.021)	0.344	0.969 (0.931-1.009)	0.128
Cisplatin-based chemotherapy	0.672 (0.248-1.823)	0.435	0.526 (0.193-1.432)	0.209	0.727 (0.257-2.059)	0.549	0.555 (0.189-1.629)	0.284
Surgical staging	1.308 (0.874-1.958)	0.193	1.171 (0.747-1.835)	0.492	1.382 (0.918-2.079)	0.121	1.143 (0.721-1.810)	0.570

### Treatment-related complications

Treatment-related complications are shown in **Table 4**. In CTCAE v5.0, perineal edema corresponds to Genital Edema, and lower-extremity edema to Peripheral Edema. Because of the small sample size, these two items were combined for presentation. In the surgical staging group, the incidence of grade 3/4 myelosuppression, grade 3/4 gastrointestinal disorders, and grade 3/4 perineal and lower-extremity edema was 29.9%, 9.3%, and 8.2%, respectively. In the clinical staging group, the corresponding rates were 27.8%, 8.7%, and 6.7%. None of these differences was statistically significant.

**Table 4:** Treatment-related complications.

Complications	Surgical staging group (n = 97)	Clinical staging group (n = 299)	*P* value
Grade 3/4 myelosuppression, n (%)	29 (29.9%)	83 (27.8%)	0.685
Grade 3/4 gastrointestinal disorder, n (%)	9 (9.3%)	26 (8.7%)	0.861
Grade 3/4 perineal and lower-extremity edema, n (%)	8 (8.2%)	20 (6.7%)	0.603

In CTCAE v5.0, perineal edema is classified as Edema of the Trunk and Genital Area, and lower-extremity edema as Peripheral Edema.

## Discussion

Our findings indicated that lymphadenectomy before chemoradiotherapy does not improve long-term oncologic outcomes in patients with stage IIB–IIIC cervical cancer. Multivariable Cox regression analysis confirmed that tumor histology, pelvic wall involvement, and imaging-positive pelvic and/or para-aortic lymph nodes were independent prognostic factors. In terms of surgical safety, the incidences of grade 3/4 myelosuppression, grade 3/4 gastrointestinal disorders, and grade 3/4 perineal and lower-extremity edema were similar in the surgical and clinical staging groups.

In the surgical staging group, pathological para-aortic and pelvic lymph node metastasis in cervical cancer generally follows a stepwise dissemination pattern, spreading sequentially from pelvic lymph nodes to common iliac nodes and then to para-aortic lymph nodes (24). Previous studies have shown that the obturator fossa is the most common site of pelvic lymph node metastasis, followed by the internal and external iliac nodal regions (25). Clinically, isolated para-aortic lymph node metastasis rarely occurs independently (26), and is almost exclusively secondary to pelvic lymphatic involvement (8). In the surgical staging group, no patient had isolated para-aortic lymph node metastasis without pelvic lymph node involvement. Collectively, these lymph node metastasis patterns are consistent with previous findings.

In the surgical staging group, pathologic verification showed that conventional imaging had a sensitivity of 80.0% and specificity of 72.3% for detecting lymph node metastasis. Imaging has inherent limitations in detecting lymph node metastasis, particularly occult micrometastases and non-enlarged microscopic metastatic foci. Postoperative nodal pathology may upstage patients with cervical cancer after lymph node dissection in locally advanced cervical cancer. Metastatic involvement of the common iliac and/or para-aortic lymph nodes critically determines whether EF-EBRT should encompass the para-aortic region. Researchers are attempting to improve noninvasive identification of lymph node metastasis in patients with cervical cancer. PET-CT is widely used to diagnose retroperitoneal lymph node metastasis and has a low false-negative rate but a high false-positive rate (27); therefore, correlation with other imaging modalities or biopsy is required to confirm malignancy. In addition, prediction models integrating clinicopathologic factors demonstrate high accuracy in predicting para-aortic lymph node metastasis (26, 28).

Surgical resection of para-aortic ± pelvic lymph nodes is another method of diagnosing lymph node metastasis, but its therapeutic value remains controversial. While preoperative surgical staging provides accurate pathologic nodal assessment, our findings imply that long-term oncologic benefits may not be evident with this approach in stage IIB–IIIC cervical cancer. Notably, in patients with imaging-positive lymph nodes, PFS was numerically lower in the surgical staging group than in the clinical staging group (50.9% vs. 64.0%; *p* = 0.057), without a statistically significant difference. Nevertheless, the borderline *p* value of 0.057 suggests that this finding warrants further investigation in larger prospective studies.

Our findings are consistent with some previous reports. Two large-scale cohort studies found that para-aortic lymphadenectomy was not associated with a survival benefit (29, 30). Three other retrospective studies confirmed no significant differences in PFS and OS between surgical staging and chemoradiotherapy (13, 31, 32). Uterus-11, a multicenter prospective randomized phase III trial, found no difference in disease-free survival between surgical and clinical staging in patients with locally advanced cervical cancer, but a significant disease-free survival benefit in patients with stage IIB cervical cancer (12). Multiple studies have shown that surgical staging improves the prognosis of selected patients with cervical cancer; however, it remains unclear whether this benefit extends to patients with imaging-positive or imaging-negative lymph nodes. Two additional large cohort studies support improved outcomes with surgical staging in patients with negative imaging findings (16, 17). Another retrospective study reported improved 5-year PFS and OS in patients with pelvic lymph nodes ≥ 1.5 cm in diameter or multiple imaging-positive pelvic lymph nodes (18). In a retrospective cohort study by Pomel et al., para-aortic surgical staging in locally advanced cervical cancer was more deleterious for patients in terms of OS and PFS than clinical staging (14).

These findings further support that preoperative surgical staging does not improve long-term oncologic outcomes in stage IIB–IIIC cervical cancer, despite its ability to provide precise pathologic nodal assessment. The absence of a survival benefit may be attributable to multiple clinical and biological factors. First, lymphadenectomy removes only macroscopic metastatic lesions and cannot eliminate occult micrometastases and circulating tumor cells, which are common in stage IIB–IIIC disease and may be major sources of recurrence. Moreover, given the relatively short median interval (11 days) between surgical staging and subsequent chemoradiotherapy, any potential adverse effect related to treatment delay should be interpreted cautiously.

Our study showed that laparoscopic surgical staging did not increase the incidences of grade 3/4 myelosuppression, grade 3/4 gastrointestinal toxicities, or perineal and lower-extremity edema compared with clinical staging alone. Previous studies have shown that lymphadenectomy for cervical cancer increases the risk of lower-extremity lymphedema (33). Pelvic fibrosis induced by concurrent chemoradiotherapy is also a recognized contributor to pelvic lymphatic obstruction and lower-extremity edema, indicating that postoperative edema is multifactorial rather than a surgery-dependent adverse event. In early-stage cervical cancer, sentinel lymph node biopsy is currently recommended to replace systematic pelvic lymphadenectomy to reduce surgical trauma and lymphedema risk (34). Similarly, our results suggest that the surgical staging procedure was feasible in selected patients with adequate preoperative fitness.

Strengths and limitations: This study has several strengths. First, it had a relatively large sample size and applied strict inclusion criteria, resulting in a homogeneous patient cohort and consistent treatment regimens; this enhances the reliability of the survival analyses. Second, as a real-world investigation, it provides an objective assessment of the practical efficacy of surgical staging in routine clinical practice. This study has several important limitations. First, it is a retrospective, non-randomized comparison subject to confounding by indication; treatment allocation was not randomized, and selection bias could not be fully eliminated even after multivariate adjustment. Second, the 2018 FIGO staging revision integrated imaging and pathologic nodal status into staging and introduced stage IIIC. This staging update changed risk stratification and standard treatment recommendations and may have affected our group comparisons. Third, the sample size of patients with non-squamous cell carcinoma was relatively small, which limited the statistical power of relevant subgroup analyses; these subgroup analyses were exploratory, and firm conclusions cannot be drawn from them. Fourth, no centralized imaging review was performed; imaging interpretations relied on local radiologists, and inter-reader variability may exist. Finally, we did not have complete medical records to quantify how often surgical pathological findings modified the radiotherapy field or subsequent treatment strategy. Therefore, we cannot evaluate the clinical impact of surgical staging on treatment adjustment, which limits the interpretation of our findings.

The findings of this real-world study offer practical guidance for assessing the value of surgical staging in cervical cancer treatment. Because invasive staging may prolong treatment time and increase medical costs without providing a survival benefit, the findings of our study do not support routine surgical staging in patients with FIGO stage IIB–IIIC cervical cancer. Further large, prospective clinical trials are warranted to validate the clinical value of surgical staging in cervical cancer and to establish more precise selection criteria.

## Conclusions

This real-world study showed that laparoscopic para-aortic and pelvic lymphadenectomy enables accurate surgical staging and has acceptable surgical safety in patients with stage IIB–IIIC cervical cancer. However, surgical staging did not improve PFS and OS. Tumor histology, pelvic wall involvement, and imaging-positive lymph nodes were the main prognostic factors.

## Data Availability

The data analyzed in this study is subject to the following licenses/restrictions: The dataset contains sensitive individual patient clinical information. Restrictions are imposed to protect patient confidentiality in accordance with local data−protection regulations. De−identified aggregated data may be obtained from the corresponding author upon reasonable request, subject to formal approval by the Ethics Committee of Chongqing University Cancer Hospital. Raw individual patient data cannot be shared publicly. Requests to access these datasets should be directed to Qin Zhang: zhangqin_020@163.com.
